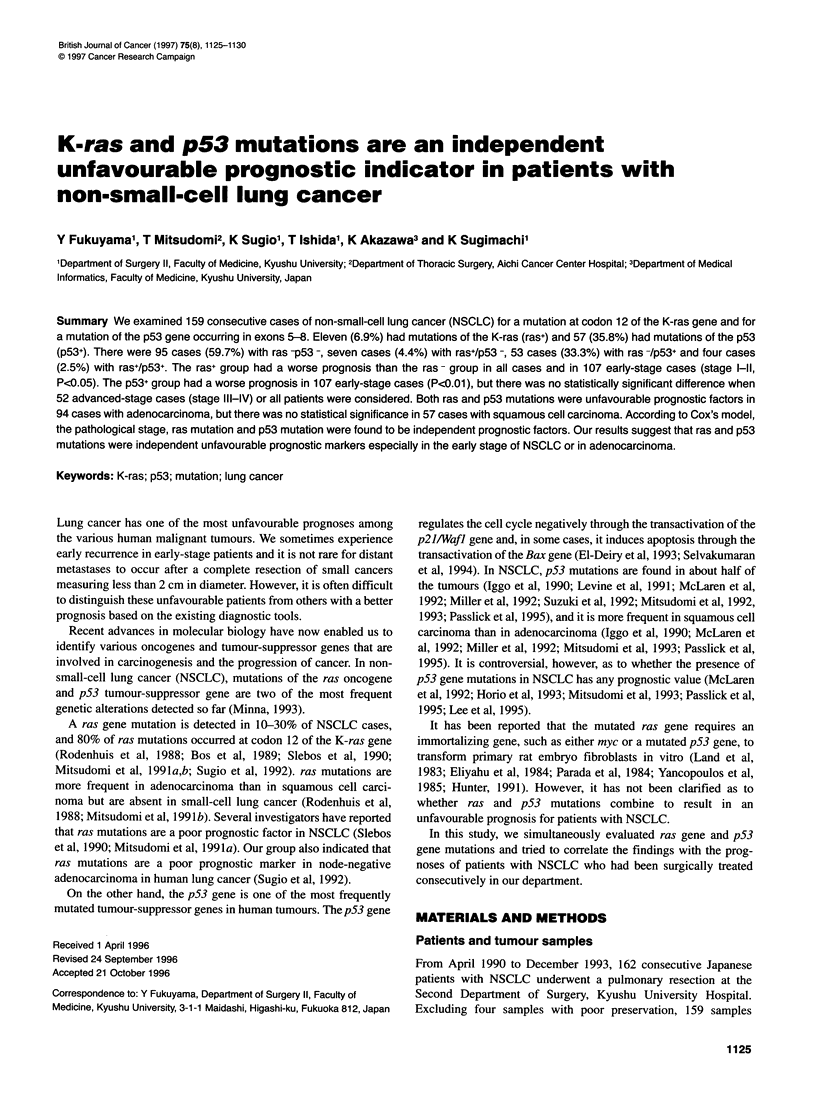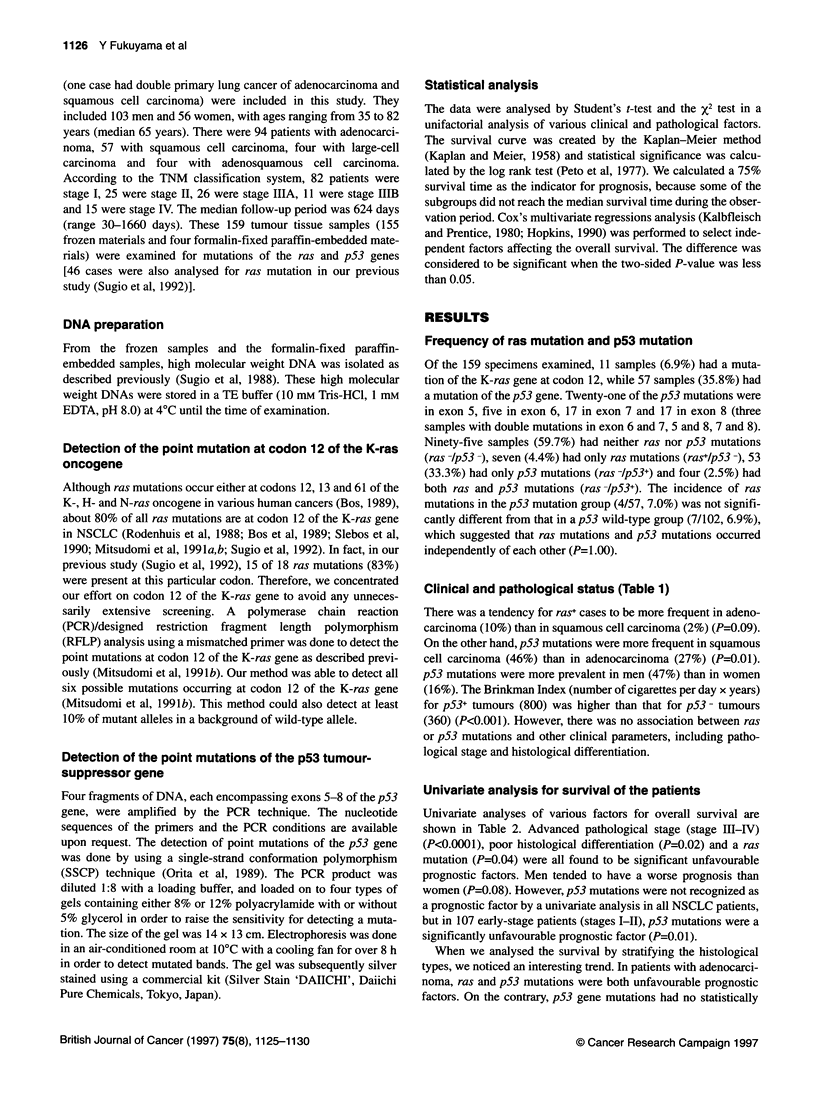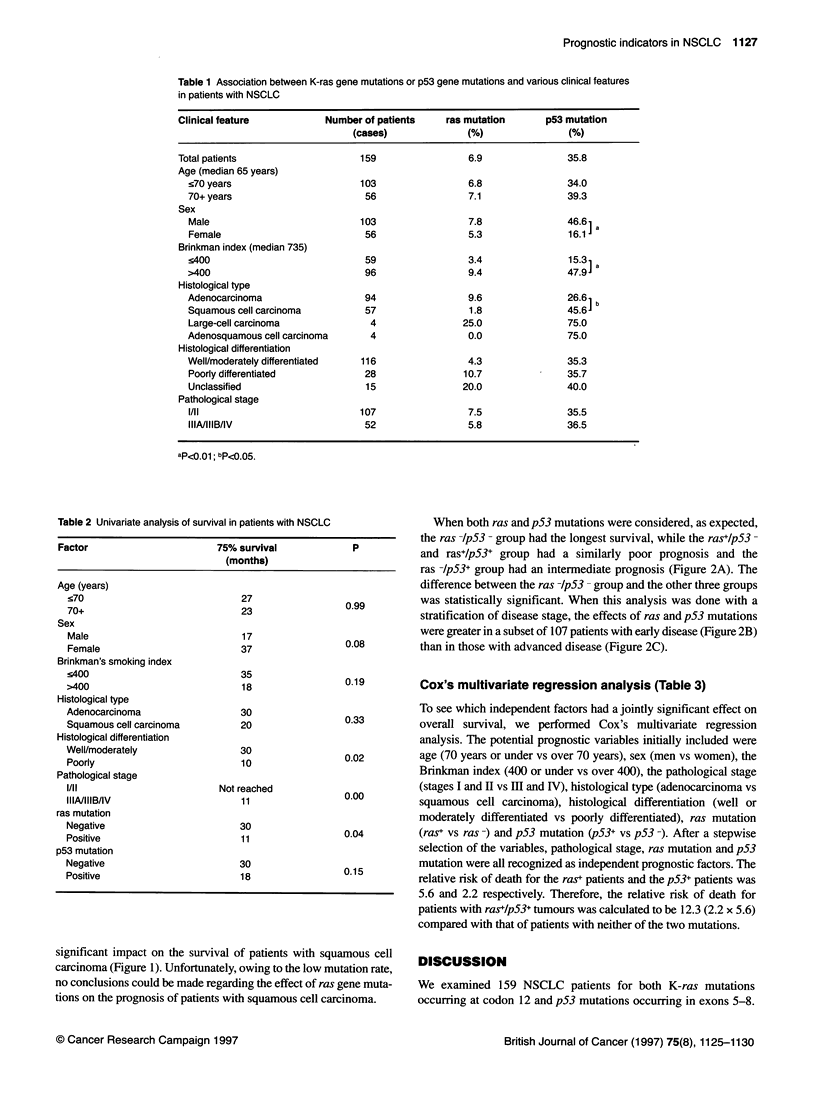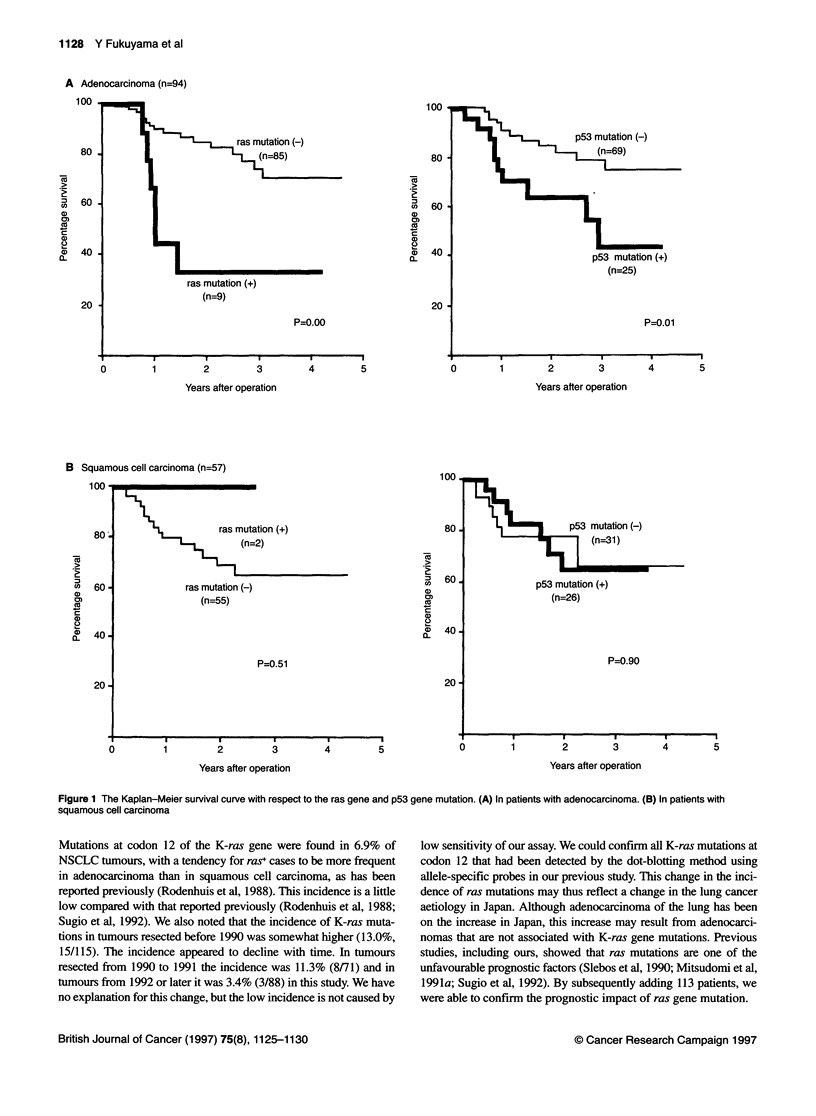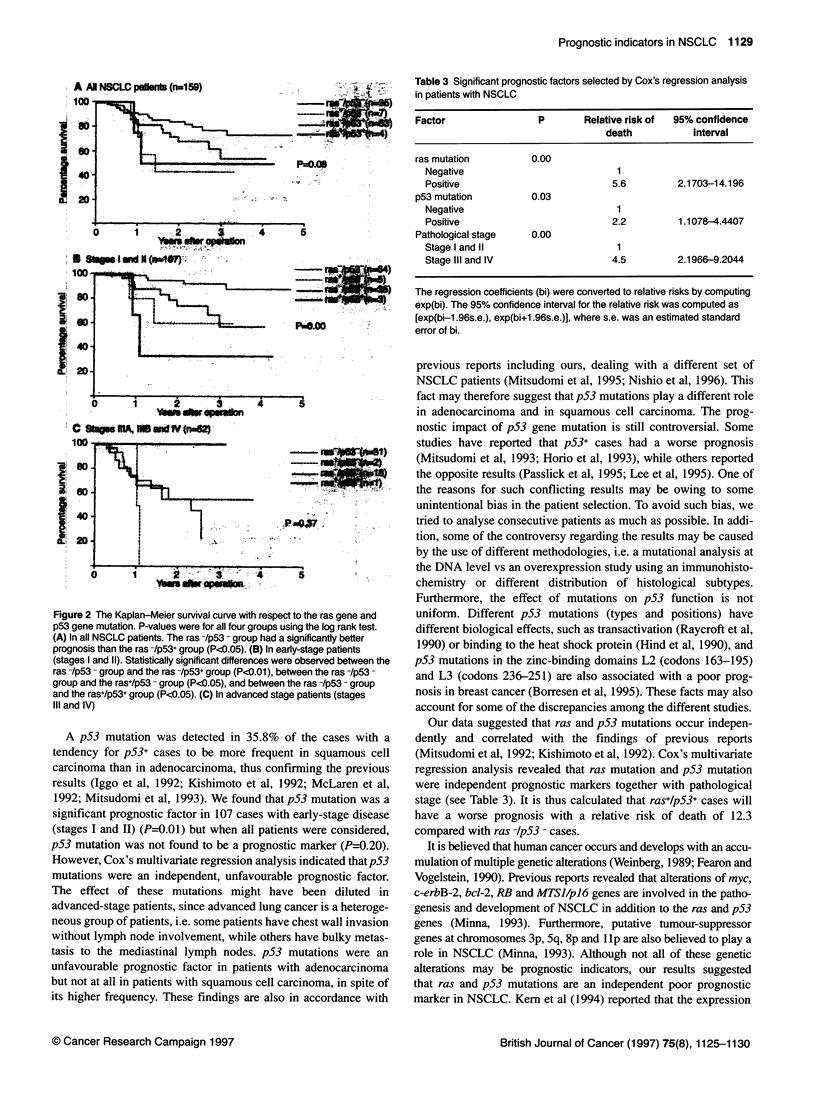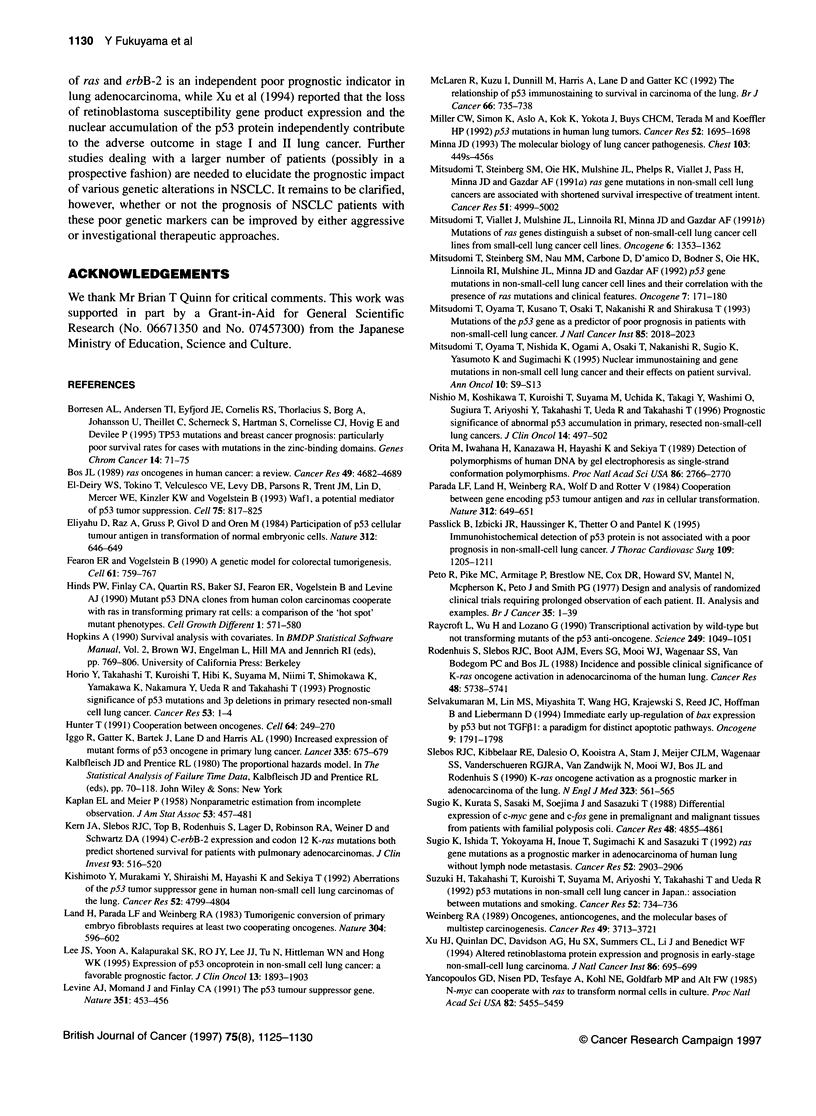# K-ras and p53 mutations are an independent unfavourable prognostic indicator in patients with non-small-cell lung cancer.

**DOI:** 10.1038/bjc.1997.194

**Published:** 1997

**Authors:** Y. Fukuyama, T. Mitsudomi, K. Sugio, T. Ishida, K. Akazawa, K. Sugimachi

**Affiliations:** Department of Surgery II, Faculty of Medicine, Kyushu University, Japan.

## Abstract

We examined 159 consecutive cases of non-small-cell lung cancer (NSCLC) for a mutation at codon 12 of the K-ras gene and for a mutation of the p53 gene occurring in exons 5-8. Eleven (6.9%) had mutations of the K-ras (ras+) and 57 (35.8%) had mutations of the p53 (p53+). There were 95 cases (59.7%) with ras- p53-, seven cases (4.4%) with ras+/p53-, 53 cases (33.3%) with ras-/p53+ and four cases (2.5%) with ras+/p53+. The ras+ group had a worse prognosis than the ras group in all cases and in 107 early-stage cases (stage I-II, P<0.05). The p53+ group had a worse prognosis in 107 early-stage cases (P<0.01), but there was no statistically significant difference when 52 advanced-stage cases (stage III-IV) or all patients were considered. Both ras and p53 mutations were unfavourable prognostic factors in 94 cases with adenocarcinoma, but there was no statistical significance in 57 cases with squamous cell carcinoma. According to Cox's model, the pathological stage, ras mutation and p53 mutation were found to be independent prognostic factors. Our results suggest that ras and p53 mutations were independent unfavourable prognostic markers especially in the early stage of NSCLC or in adenocarcinoma.


British Journal of Cancer (1997) 75(8), 1125–1130
© 1997 Cancer Research Campaign

### K-*ras* and *p53* mutations are an independent unfavourable prognostic indicator in patients with non-small-cell lung cancer

**Y Fukuyama[1], T Mitsudomi[2], K Sugio[1], T Ishida[1], K Akazawa[3] and K Sugimachi[1]**

[1]Department of Surgery II, Faculty of Medicine, Kyushu University; [2]Department of Thoracic Surgery, Aichi Cancer Center Hospital; [3]Department of Medical Informatics, Faculty of Medicine, Kyushu University, Japan

**Summary** We examined 159 consecutive cases of non-small-cell lung cancer (NSCLC) for a mutation at codon 12 of the K-ras gene and for a mutation of the p53 gene occurring in exons 5–8. Eleven (6.9%) had mutations of the K-ras (ras$^+$) and 57 (35.8%) had mutations of the p53 (p53$^+$). There were 95 cases (59.7%) with ras$^-$p53$^-$, seven cases (4.4%) with ras$^+$/p53$^-$, 53 cases (33.3%) with ras$^-$/p53$^+$ and four cases (2.5%) with ras$^+$/p53$^+$. The ras$^+$ group had a worse prognosis than the ras$^-$ group in all cases and in 107 early-stage cases (stage I–II, $P<0.05$). The p53$^+$ group had a worse prognosis in 107 early-stage cases ($P<0.01$), but there was no statistically significant difference when 52 advanced-stage cases (stage III–IV) or all patients were considered. Both ras and p53 mutations were unfavourable prognostic factors in 94 cases with adenocarcinoma, but there was no statistical significance in 57 cases with squamous cell carcinoma. According to Cox's model, the pathological stage, ras mutation and p53 mutation were found to be independent prognostic factors. Our results suggest that ras and p53 mutations were independent unfavourable prognostic markers especially in the early stage of NSCLC or in adenocarcinoma.

**Keywords:** K-ras; p53; mutation; lung cancer

Lung cancer has one of the most unfavourable prognoses among the various human malignant tumours. We sometimes experience early recurrence in early-stage patients and it is not rare for distant metastases to occur after a complete resection of small cancers measuring less than 2 cm in diameter. However, it is often difficult to distinguish these unfavourable patients from others with a better prognosis based on the existing diagnostic tools.

Recent advances in molecular biology have now enabled us to identify various oncogenes and tumour-suppressor genes that are involved in carcinogenesis and the progression of cancer. In non-small-cell lung cancer (NSCLC), mutations of the *ras* oncogene and *p53* tumour-suppressor gene are two of the most frequent genetic alterations detected so far (Minna, 1993).

A *ras* gene mutation is detected in 10–30% of NSCLC cases, and 80% of *ras* mutations occurred at codon 12 of the K-*ras* gene (Rodenhuis et al, 1988; Bos et al, 1989; Slebos et al, 1990; Mitsudomi et al, 1991*a,b*; Sugio et al, 1992). *ras* mutations are more frequent in adenocarcinoma than in squamous cell carcinoma but are absent in small-cell lung cancer (Rodenhuis et al, 1988; Mitsudomi et al, 1991*b*). Several investigators have reported that *ras* mutations are a poor prognostic factor in NSCLC (Slebos et al, 1990; Mitsudomi et al, 1991*a*). Our group also indicated that *ras* mutations are a poor prognostic marker in node-negative adenocarcinoma in human lung cancer (Sugio et al, 1992).

On the other hand, the *p53* gene is one of the most frequently mutated tumour-suppressor genes in human tumours. The *p53* gene regulates the cell cycle negatively through the transactivation of the *p21/Waf1* gene and, in some cases, it induces apoptosis through the transactivation of the *Bax* gene (El-Deiry et al, 1993; Selvakumaran et al, 1994). In NSCLC, *p53* mutations are found in about half of the tumours (Iggo et al, 1990; Levine et al, 1991; McLaren et al, 1992; Miller et al, 1992; Suzuki et al, 1992; Mitsudomi et al, 1992, 1993; Passlick et al, 1995), and it is more frequent in squamous cell carcinoma than in adenocarcinoma (Iggo et al, 1990; McLaren et al, 1992; Miller et al, 1992; Mitsudomi et al, 1993; Passlick et al, 1995). It is controversial, however, as to whether the presence of *p53* gene mutations in NSCLC has any prognostic value (McLaren et al, 1992; Horio et al, 1993; Mitsudomi et al, 1993; Passlick et al, 1995; Lee et al, 1995).

It has been reported that the mutated *ras* gene requires an immortalizing gene, such as either *myc* or a mutated *p53* gene, to transform primary rat embryo fibroblasts in vitro (Land et al, 1983; Eliyahu et al, 1984; Parada et al, 1984; Yancopoulos et al, 1985; Hunter, 1991). However, it has not been clarified as to whether *ras* and *p53* mutations combine to result in an unfavourable prognosis for patients with NSCLC.

In this study, we simultaneously evaluated *ras* gene and *p53* gene mutations and tried to correlate the findings with the prognoses of patients with NSCLC who had been surgically treated consecutively in our department.

Received 1 April 1996
Revised 24 September 1996
Accepted 21 October 1996

Correspondence to: Y Fukuyama, Department of Surgery II, Faculty of Medicine, Kyushu University, 3-1-1 Maidashi, Higashi-ku, Fukuoka 812, Japan

### MATERIALS AND METHODS

### Patients and tumour samples

From April 1990 to December 1993, 162 consecutive Japanese patients with NSCLC underwent a pulmonary resection at the Second Department of Surgery, Kyushu University Hospital. Excluding four samples with poor preservation, 159 samples

(one case had double primary lung cancer of adenocarcinoma and squamous cell carcinoma) were included in this study. They included 103 men and 56 women, with ages ranging from 35 to 82 years (median 65 years). There were 94 patients with adenocarcinoma, 57 with squamous cell carcinoma, four with large-cell carcinoma and four with adenosquamous cell carcinoma. According to the TNM classification system, 82 patients were stage I, 25 were stage II, 26 were stage IIIA, 11 were stage IIIB and 15 were stage IV. The median follow-up period was 624 days (range 30–1660 days). These 159 tumour tissue samples (155 frozen materials and four formalin-fixed paraffin-embedded materials) were examined for mutations of the *ras* and *p53* genes [46 cases were also analysed for *ras* mutation in our previous study (Sugio et al, 1992)].

### DNA preparation

From the frozen samples and the formalin-fixed paraffin-embedded samples, high molecular weight DNA was isolated as described previously (Sugio et al, 1988). These high molecular weight DNAs were stored in a TE buffer (10 mM Tris-HCl, 1 mM EDTA, pH 8.0) at 4°C until the time of examination.

### Detection of the point mutation at codon 12 of the K-ras oncogene

Although *ras* mutations occur either at codons 12, 13 and 61 of the K-, H- and N-*ras* oncogene in various human cancers (Bos, 1989), about 80% of all *ras* mutations are at codon 12 of the K-*ras* gene in NSCLC (Rodenhuis et al, 1988; Bos et al, 1989; Slebos et al, 1990; Mitsudomi et al, 1991*a,b*; Sugio et al, 1992). In fact, in our previous study (Sugio et al, 1992), 15 of 18 *ras* mutations (83%) were present at this particular codon. Therefore, we concentrated our effort on codon 12 of the K-*ras* gene to avoid any unnecessarily extensive screening. A polymerase chain reaction (PCR)/designed restriction fragment length polymorphism (RFLP) analysis using a mismatched primer was done to detect the point mutations at codon 12 of the K-*ras* gene as described previously (Mitsudomi et al, 1991*b*). Our method was able to detect all six possible mutations occurring at codon 12 of the K-*ras* gene (Mitsudomi et al, 1991*b*). This method could also detect at least 10% of mutant alleles in a background of wild-type allele.

### Detection of the point mutations of the p53 tumour-suppressor gene

Four fragments of DNA, each encompassing exons 5–8 of the *p53* gene, were amplified by the PCR technique. The nucleotide sequences of the primers and the PCR conditions are available upon request. The detection of point mutations of the *p53* gene was done by using a single-strand conformation polymorphism (SSCP) technique (Orita et al, 1989). The PCR product was diluted 1:8 with a loading buffer, and loaded on to four types of gels containing either 8% or 12% polyacrylamide with or without 5% glycerol in order to raise the sensitivity for detecting a mutation. The size of the gel was 14 × 13 cm. Electrophoresis was done in an air-conditioned room at 10°C with a cooling fan for over 8 h in order to detect mutated bands. The gel was subsequently silver stained using a commercial kit (Silver Stain 'DAIICHI', Daiichi Pure Chemicals, Tokyo, Japan).

### Statistical analysis

The data were analysed by Student's *t*-test and the $\chi^2$ test in a unifactorial analysis of various clinical and pathological factors. The survival curve was created by the Kaplan–Meier method (Kaplan and Meier, 1958) and statistical significance was calculated by the log rank test (Peto et al, 1977). We calculated a 75% survival time as the indicator for prognosis, because some of the subgroups did not reach the median survival time during the observation period. Cox's multivariate regressions analysis (Kalbfleisch and Prentice, 1980; Hopkins, 1990) was performed to select independent factors affecting the overall survival. The difference was considered to be significant when the two-sided *P*-value was less than 0.05.

### RESULTS

### Frequency of ras mutation and p53 mutation

Of the 159 specimens examined, 11 samples (6.9%) had a mutation of the K-*ras* gene at codon 12, while 57 samples (35.8%) had a mutation of the *p53* gene. Twenty-one of the *p53* mutations were in exon 5, five in exon 6, 17 in exon 7 and 17 in exon 8 (three samples with double mutations in exon 6 and 7, 5 and 8, 7 and 8). Ninety-five samples (59.7%) had neither *ras* nor *p53* mutations ($ras^-/p53^-$), seven (4.4%) had only *ras* mutations ($ras^+/p53^-$), 53 (33.3%) had only *p53* mutations ($ras^-/p53^+$) and four (2.5%) had both *ras* and *p53* mutations ($ras^-/p53^+$). The incidence of *ras* mutations in the *p53* mutation group (4/57, 7.0%) was not significantly different from that in a *p53* wild-type group (7/102, 6.9%), which suggested that *ras* mutations and *p53* mutations occurred independently of each other ($P$=1.00).

### Clinical and pathological status (Table 1)

There was a tendency for $ras^+$ cases to be more frequent in adenocarcinoma (10%) than in squamous cell carcinoma (2%) ($P$=0.09). On the other hand, *p53* mutations were more frequent in squamous cell carcinoma (46%) than in adenocarcinoma (27%) ($P$=0.01). *p53* mutations were more prevalent in men (47%) than in women (16%). The Brinkman Index (number of cigarettes per day × years) for $p53^+$ tumours (800) was higher than that for $p53^-$ tumours (360) ($P$<0.001). However, there was no association between *ras* or *p53* mutations and other clinical parameters, including pathological stage and histological differentiation.

### Univariate analysis for survival of the patients

Univariate analyses of various factors for overall survival are shown in Table 2. Advanced pathological stage (stage III–IV) ($P$<0.0001), poor histological differentiation ($P$=0.02) and a *ras* mutation ($P$=0.04) were all found to be significant unfavourable prognostic factors. Men tended to have a worse prognosis than women ($P$=0.08). However, *p53* mutations were not recognized as a prognostic factor by a univariate analysis in all NSCLC patients, but in 107 early-stage patients (stages I–II), *p53* mutations were a significantly unfavourable prognostic factor ($P$=0.01).

When we analysed the survival by stratifying the histological types, we noticed an interesting trend. In patients with adenocarcinoma, *ras* and *p53* mutations were both unfavourable prognostic factors. On the contrary, *p53* gene mutations had no statistically

© Cancer Research Campaign 1997

**Table 1** Association between K-ras gene mutations or p53 gene mutations and various clinical features in patients with NSCLC

| Clinical feature | Number of patients (cases) | ras mutation (%) | p53 mutation (%) | |
|---|---|---|---|---|
| Total patients | 159 | 6.9 | 35.8 | |
| Age (median 65 years) | | | | |
| ≤70 years | 103 | 6.8 | 34.0 | |
| 70+ years | 56 | 7.1 | 39.3 | |
| Sex | | | | |
| Male | 103 | 7.8 | 46.6 | ]a |
| Female | 56 | 5.3 | 16.1 | |
| Brinkman index (median 735) | | | | |
| ≤400 | 59 | 3.4 | 15.3 | ]a |
| >400 | 96 | 9.4 | 47.9 | |
| Histological type | | | | |
| Adenocarcinoma | 94 | 9.6 | 26.6 | ]b |
| Squamous cell carcinoma | 57 | 1.8 | 45.6 | |
| Large-cell carcinoma | 4 | 25.0 | 75.0 | |
| Adenosquamous cell carcinoma | 4 | 0.0 | 75.0 | |
| Histological differentiation | | | | |
| Well/moderately differentiated | 116 | 4.3 | 35.3 | |
| Poorly differentiated | 28 | 10.7 | 35.7 | |
| Unclassified | 15 | 20.0 | 40.0 | |
| Pathological stage | | | | |
| I/II | 107 | 7.5 | 35.5 | |
| IIIA/IIIB/IV | 52 | 5.8 | 36.5 | |

[a]$P<0.01$; [b]$P<0.05$.

**Table 2** Univariate analysis of survival in patients with NSCLC

| Factor | 75% survival (months) | P |
|---|---|---|
| Age (years) | | |
| ≤70 | 27 | 0.99 |
| 70+ | 23 | |
| Sex | | |
| Male | 17 | 0.08 |
| Female | 37 | |
| Brinkman's smoking index | | |
| ≤400 | 35 | 0.19 |
| >400 | 18 | |
| Histological type | | |
| Adenocarcinoma | 30 | 0.33 |
| Squamous cell carcinoma | 20 | |
| Histological differentiation | | |
| Well/moderately | 30 | 0.02 |
| Poorly | 10 | |
| Pathological stage | | |
| I/II | Not reached | 0.00 |
| IIIA/IIIB/IV | 11 | |
| ras mutation | | |
| Negative | 30 | 0.04 |
| Positive | 11 | |
| p53 mutation | | |
| Negative | 30 | 0.15 |
| Positive | 18 | |

significant impact on the survival of patients with squamous cell carcinoma (Figure 1). Unfortunately, owing to the low mutation rate, no conclusions could be made regarding the effect of *ras* gene mutations on the prognosis of patients with squamous cell carcinoma.

When both *ras* and *p53* mutations were considered, as expected, the $ras^-/p53^-$ group had the longest survival, while the $ras^+/p53^-$ and $ras^+/p53^+$ group had a similarly poor prognosis and the $ras^-/p53^+$ group had an intermediate prognosis (Figure 2A). The difference between the $ras^-/p53^-$ group and the other three groups was statistically significant. When this analysis was done with a stratification of disease stage, the effects of *ras* and *p53* mutations were greater in a subset of 107 patients with early disease (Figure 2B) than in those with advanced disease (Figure 2C).

### Cox's multivariate regression analysis (Table 3)

To see which independent factors had a jointly significant effect on overall survival, we performed Cox's multivariate regression analysis. The potential prognostic variables initially included were age (70 years or under vs over 70 years), sex (men vs women), the Brinkman index (400 or under vs over 400), the pathological stage (stages I and II vs III and IV), histological type (adenocarcinoma vs squamous cell carcinoma), histological differentiation (well or moderately differentiated vs poorly differentiated), *ras* mutation ($ras^+$ vs $ras^-$) and *p53* mutation ($p53^+$ vs $p53^-$). After a stepwise selection of the variables, pathological stage, *ras* mutation and *p53* mutation were all recognized as independent prognostic factors. The relative risk of death for the $ras^+$ patients and the $p53^+$ patients was 5.6 and 2.2 respectively. Therefore, the relative risk of death for patients with $ras^+/p53^+$ tumours was calculated to be 12.3 ($2.2 \times 5.6$) compared with that of patients with neither of the two mutations.

### DISCUSSION

We examined 159 NSCLC patients for both K-*ras* mutations occurring at codon 12 and *p53* mutations occurring in exons 5–8.

© Cancer Research Campaign 1997

**Figure 1** The Kaplan–Meier survival curve with respect to the ras gene and p53 gene mutation. **(A)** In patients with adenocarcinoma. **(B)** In patients with squamous cell carcinoma

Mutations at codon 12 of the K-*ras* gene were found in 6.9% of NSCLC tumours, with a tendency for *ras*$^+$ cases to be more frequent in adenocarcinoma than in squamous cell carcinoma, as has been reported previously (Rodenhuis et al, 1988). This incidence is a little low compared with that reported previously (Rodenhuis et al, 1988; Sugio et al, 1992). We also noted that the incidence of K-*ras* mutations in tumours resected before 1990 was somewhat higher (13.0%, 15/115). The incidence appeared to decline with time. In tumours resected from 1990 to 1991 the incidence was 11.3% (8/71) and in tumours from 1992 or later it was 3.4% (3/88) in this study. We have no explanation for this change, but the low incidence is not caused by low sensitivity of our assay. We could confirm all K-*ras* mutations at codon 12 that had been detected by the dot-blotting method using allele-specific probes in our previous study. This change in the incidence of *ras* mutations may thus reflect a change in the lung cancer aetiology in Japan. Although adenocarcinoma of the lung has been on the increase in Japan, this increase may result from adenocarcinomas that are not associated with K-*ras* gene mutations. Previous studies, including ours, showed that *ras* mutations are one of the unfavourable prognostic factors (Slebos et al, 1990; Mitsudomi et al, 1991*a*; Sugio et al, 1992). By subsequently adding 113 patients, we were able to confirm the prognostic impact of *ras* gene mutation.

© Cancer Research Campaign 1997

**Figure 2** The Kaplan–Meier survival curve with respect to the ras gene and p53 gene mutation. P-values were for all four groups using the log rank test. **(A)** In all NSCLC patients. The ras $^-$/p53 $^-$ group had a significantly better prognosis than the ras $^-$/p53$^+$ group (P<0.05). **(B)** In early-stage patients (stages I and II). Statistically significant differences were observed between the ras $^-$/p53 $^-$ group and the ras $^-$/p53$^+$ group (P<0.01), between the ras $^-$/p53 $^-$ group and the ras$^+$/p53 $^-$ group (P<0.05), and between the ras $^-$/p53 $^-$ group and the ras$^+$/p53$^+$ group (P<0.05). **(C)** In advanced stage patients (stages III and IV)

A *p53* mutation was detected in 35.8% of the cases with a tendency for *p53*$^+$ cases to be more frequent in squamous cell carcinoma than in adenocarcinoma, thus confirming the previous results (Iggo et al, 1992; Kishimoto et al, 1992; McLaren et al, 1992; Mitsudomi et al, 1993). We found that *p53* mutation was a significant prognostic factor in 107 cases with early-stage disease (stages I and II) (*P*=0.01) but when all patients were considered, *p53* mutation was not found to be a prognostic marker (*P*=0.20). However, Cox's multivariate regression analysis indicated that *p53* mutations were an independent, unfavourable prognostic factor. The effect of these mutations might have been diluted in advanced-stage patients, since advanced lung cancer is a heterogeneous group of patients, i.e. some patients have chest wall invasion without lymph node involvement, while others have bulky metastasis to the mediastinal lymph nodes. *p53* mutations were an unfavourable prognostic factor in patients with adenocarcinoma but not at all in patients with squamous cell carcinoma, in spite of its higher frequency. These findings are also in accordance with previous reports including ours, dealing with a different set of NSCLC patients (Mitsudomi et al, 1995; Nishio et al, 1996). This fact may therefore suggest that *p53* mutations play a different role in adenocarcinoma and in squamous cell carcinoma. The prognostic impact of *p53* gene mutation is still controversial. Some studies have reported that *p53*$^+$ cases had a worse prognosis (Mitsudomi et al, 1993; Horio et al, 1993), while others reported the opposite results (Passlick et al, 1995; Lee et al, 1995). One of the reasons for such conflicting results may be owing to some unintentional bias in the patient selection. To avoid such bias, we tried to analyse consecutive patients as much as possible. In addition, some of the controversy regarding the results may be caused by the use of different methodologies, i.e. a mutational analysis at the DNA level vs an overexpression study using an immunohistochemistry or different distribution of histological subtypes. Furthermore, the effect of mutations on *p53* function is not uniform. Different *p53* mutations (types and positions) have different biological effects, such as transactivation (Raycroft et al, 1990) or binding to the heat shock protein (Hind et al, 1990), and *p53* mutations in the zinc-binding domains L2 (codons 163–195) and L3 (codons 236–251) are also associated with a poor prognosis in breast cancer (Borresen et al, 1995). These facts may also account for some of the discrepancies among the different studies.

**Table 3** Significant prognostic factors selected by Cox's regression analysis in patients with NSCLC

| Factor | P | Relative risk of death | 95% confidence interval |
|---|---|---|---|
| ras mutation | 0.00 | | |
| Negative | | 1 | |
| Positive | | 5.6 | 2.1703–14.196 |
| p53 mutation | 0.03 | | |
| Negative | | 1 | |
| Positive | | 2.2 | 1.1078–4.4407 |
| Pathological stage | 0.00 | | |
| Stage I and II | | 1 | |
| Stage III and IV | | 4.5 | 2.1966–9.2044 |

The regression coefficients (bi) were converted to relative risks by computing exp(bi). The 95% confidence interval for the relative risk was computed as [exp(bi–1.96s.e.), exp(bi+1.96s.e.)], where s.e. was an estimated standard error of bi.

Our data suggested that *ras* and *p53* mutations occur independently and correlated with the findings of previous reports (Mitsudomi et al, 1992; Kishimoto et al, 1992). Cox's multivariate regression analysis revealed that *ras* mutation and *p53* mutation were independent prognostic markers together with pathological stage (see Table 3). It is thus calculated that *ras*$^+$/*p53*$^+$ cases will have a worse prognosis with a relative risk of death of 12.3 compared with *ras* $^-$/*p53* $^-$ cases.

It is believed that human cancer occurs and develops with an accumulation of multiple genetic alterations (Weinberg, 1989; Fearon and Vogelstein, 1990). Previous reports revealed that alterations of *myc*, c-*erb*B-2, *bcl*-2, *RB* and *MTS1/p16* genes are involved in the pathogenesis and development of NSCLC in addition to the *ras* and *p53* genes (Minna, 1993). Furthermore, putative tumour-suppressor genes at chromosomes 3p, 5q, 8p and 11p are also believed to play a role in NSCLC (Minna, 1993). Although not all of these genetic alterations may be prognostic indicators, our results suggested that *ras* and *p53* mutations are an independent poor prognostic marker in NSCLC. Kern et al (1994) reported that the expression

© Cancer Research Campaign 1997

of *ras* and *erb*B-2 is an independent poor prognostic indicator in lung adenocarcinoma, while Xu et al (1994) reported that the loss of retinoblastoma susceptibility gene product expression and the nuclear accumulation of the p53 protein independently contribute to the adverse outcome in stage I and II lung cancer. Further studies dealing with a larger number of patients (possibly in a prospective fashion) are needed to elucidate the prognostic impact of various genetic alterations in NSCLC. It remains to be clarified, however, whether or not the prognosis of NSCLC patients with these poor genetic markers can be improved by either aggressive or investigational therapeutic approaches.

### ACKNOWLEDGEMENTS

We thank Mr Brian T Quinn for critical comments. This work was supported in part by a Grant-in-Aid for General Scientific Research (No. 06671350 and No. 07457300) from the Japanese Ministry of Education, Science and Culture.

### REFERENCES

Borresen AL, Andersen TI, Eyfjord JE, Cornelis RS, Thorlacius S, Borg A, Johansson U, Theillet C, Scherneck S, Hartman S, Cornelisse CJ, Hovig E and Devilee P (1995) TP53 mutations and breast cancer prognosis: particularly poor survival rates for cases with mutations in the zinc-binding domains. *Genes Chrom Cancer* **14**: 71–75

Bos JL (1989) *ras* oncogenes in human cancer: a review. *Cancer Res* **49**: 4682–4689

El-Deiry WS, Tokino T, Velculesco VE, Levy DB, Parsons R, Trent JM, Lin D, Mercer WE, Kinzler KW and Vogelstein B (1993) Waf1, a potential mediator of p53 tumor suppression. *Cell* **75**: 817–825

Eliyahu D, Raz A, Gruss P, Givol D and Oren M (1984) Participation of p53 cellular tumour antigen in transformation of normal embryonic cells. *Nature* **312**: 646–649

Fearon ER and Vogelstein B (1990) A genetic model for colorectal tumorigenesis. *Cell* **61**: 759–767

Hinds PW, Finlay CA, Quartin RS, Baker SJ, Fearon ER, Vogelstein B and Levine AJ (1990) Mutant p53 DNA clones from human colon carcinomas cooperate with ras in transforming primary rat cells: a comparison of the 'hot spot' mutant phenotypes. *Cell Growth Different* **1**: 571–580

Hopkins A (1990) Survival analysis with covariates. In *BMDP Statistical Software Manual*, Vol. 2, Brown WJ, Engelman L, Hill MA and Jennrich RI (eds), pp. 769–806. University of California Press: Berkeley

Horio Y, Takahashi T, Kuroishi T, Hibi K, Suyama M, Niimi T, Shimokawa K, Yamakawa K, Nakamura Y, Ueda R and Takahashi T (1993) Prognostic significance of p53 mutations and 3p deletions in primary resected non-small cell lung cancer. *Cancer Res* **53**: 1–4

Hunter T (1991) Cooperation between oncogenes. *Cell* **64**: 249–270

Iggo R, Gatter K, Bartek J, Lane D and Harris AL (1990) Increased expression of mutant forms of p53 oncogene in primary lung cancer. *Lancet* **335**: 675–679

Kalbfleisch JD and Prentice RL (1980) The proportional hazards model. In *The Statistical Analysis of Failure Time Data*, Kalbfleisch JD and Prentice RL (eds), pp. 70–118. John Wiley & Sons: New York

Kaplan EL and Meier P (1958) Nonparametric estimation from incomplete observation. *J Am Stat Assoc* **53**: 457–481

Kern JA, Slebos RJC, Top B, Rodenhuis S, Lager D, Robinson RA, Weiner D and Schwartz DA (1994) C-*erb*B-2 expression and codon 12 K-*ras* mutations both predict shortened survival for patients with pulmonary adenocarcinomas. *J Clin Invest* **93**: 516–520

Kishimoto Y, Murakami Y, Shiraishi M, Hayashi K and Sekiya T (1992) Aberrations of the *p53* tumor suppressor gene in human non-small cell lung carcinomas of the lung. *Cancer Res* **52**: 4799–4804

Land H, Parada LF and Weinberg RA (1983) Tumorigenic conversion of primary embryo fibroblasts requires at least two cooperating oncogenes. *Nature* **304**: 596–602

Lee JS, Yoon A, Kalapurakal SK, RO JY, Lee JJ, Tu N, Hittleman WN and Hong WK (1995) Expression of p53 oncoprotein in non-small cell lung cancer: a favorable prognostic factor. *J Clin Oncol* **13**: 1893–1903

Levine AJ, Momand J and Finlay CA (1991) The p53 tumour suppressor gene. *Nature* **351**: 453–456

McLaren R, Kuzu I, Dunnill M, Harris A, Lane D and Gatter KC (1992) The relationship of p53 immunostaining to survival in carcinoma of the lung. *Br J Cancer* **66**: 735–738

Miller CW, Simon K, Aslo A, Kok K, Yokota J, Buys CHCM, Terada M and Koeffler HP (1992) *p53* mutations in human lung tumors. *Cancer Res* **52**: 1695–1698

Minna JD (1993) The molecular biology of lung cancer pathogenesis. *Chest* **103**: 449s–456s

Mitsudomi T, Steinberg SM, Oie HK, Mulshine JL, Phelps R, Viallet J, Pass H, Minna JD and Gazdar AF (1991*a*) *ras* gene mutations in non-small cell lung cancers are associated with shortened survival irrespective of treatment intent. *Cancer Res* **51**: 4999–5002

Mitsudomi T, Viallet J, Mulshine JL, Linnoila RI, Minna JD and Gazdar AF (1991*b*) Mutations of *ras* genes distinguish a subset of non-small-cell lung cancer cell lines from small-cell lung cancer cell lines. *Oncogene* **6**: 1353–1362

Mitsudomi T, Steinberg SM, Nau MM, Carbone D, D'amico D, Bodner S, Oie HK, Linnoila RI, Mulshine JL, Minna JD and Gazdar AF (1992) *p53* gene mutations in non-small-cell lung cancer cell lines and their correlation with the presence of *ras* mutations and clinical features. *Oncogene* **7**: 171–180

Mitsudomi T, Oyama T, Kusano T, Osaki T, Nakanishi R and Shirakusa T (1993) Mutations of the *p53* gene as a predictor of poor prognosis in patients with non-small-cell lung cancer. *J Natl Cancer Inst* **85**: 2018–2023

Mitsudomi T, Oyama T, Nishida K, Ogami A, Osaki T, Nakanishi R, Sugio K, Yasumoto K and Sugimachi K (1995) Nuclear immunostaining and gene mutations in non-small cell lung cancer and their effects on patient survival. *Ann Oncol* **10**: S9–S13

Nishio M, Koshikawa T, Kuroishi T, Suyama M, Uchida K, Takagi Y, Washimi O, Sugiura T, Ariyoshi Y, Takahashi T, Ueda R and Takahashi T (1996) Prognostic significance of abnormal p53 accumulation in primary, resected non-small-cell lung cancers. *J Clin Oncol* **14**: 497–502

Orita M, Iwahana H, Kanazawa H, Hayashi K and Sekiya T (1989) Detection of polymorphisms of human DNA by gel electrophoresis as single-strand conformation polymorphisms. *Proc Natl Acad Sci USA* **86**: 2766–2770

Parada LF, Land H, Weinberg RA, Wolf D and Rotter V (1984) Cooperation between gene encoding p53 tumour antigen and *ras* in cellular transformation. *Nature* **312**: 649–651

Passlick B, Izbicki JR, Haussinger K, Thetter O and Pantel K (1995) Immunohistochemical detection of p53 protein is not associated with a poor prognosis in non-small-cell lung cancer. *J Thorac Cardiovasc Surg* **109**: 1205–1211

Peto R, Pike MC, Armitage P, Brestlow NE, Cox DR, Howard SV, Mantel N, Mcpherson K, Peto J and Smith PG (1977) Design and analysis of randomized clinical trials requiring prolonged observation of each patient. II. Analysis and examples. *Br J Cancer* **35**: 1–39

Raycroft L, Wu H and Lozano G (1990) Transcriptional activation by wild-type but not transforming mutants of the p53 anti-oncogene. *Science* **249**: 1049–1051

Rodenhuis S, Slebos RJC, Boot AJM, Evers SG, Mooi WJ, Wagenaar SS, Van Bodegom PC and Bos JL (1988) Incidence and possible clinical significance of K-*ras* oncogene activation in adenocarcinoma of the human lung. *Cancer Res* **48**: 5738–5741

Selvakumaran M, Lin MS, Miyashita T, Wang HG, Krajewski S, Reed JC, Hoffman B and Liebermann D (1994) Immediate early up-regulation of *bax* expression by p53 but not TGF$\beta$1: a paradigm for distinct apoptotic pathways. *Oncogene* **9**: 1791–1798

Slebos RJC, Kibbelaar RE, Dalesio O, Kooistra A, Stam J, Meijer CJLM, Wagenaar SS, Vanderschueren RGJRA, Van Zandwijk N, Mooi WJ, Bos JL and Rodenhuis S (1990) K-*ras* oncogene activation as a prognostic marker in adenocarcinoma of the lung. *N Engl J Med* **323**: 561–565

Sugio K, Kurata S, Sasaki M, Soejima J and Sasazuki T (1988) Differential expression of c-*myc* gene and c-*fos* gene in premalignant and malignant tissues from patients with familial polyposis coli. *Cancer Res* **48**: 4855–4861

Sugio K, Ishida T, Yokoyama H, Inoue T, Sugimachi K and Sasazuki T (1992) *ras* gene mutations as a prognostic marker in adenocarcinoma of human lung without lymph node metastasis. *Cancer Res* **52**: 2903–2906

Suzuki H, Takahashi T, Kuroishi T, Suyama M, Ariyoshi Y, Takahashi T and Ueda R (1992) p53 mutations in non-small cell lung cancer in Japan.: association between mutations and smoking. *Cancer Res* **52**: 734–736

Weinberg RA (1989) Oncogenes, antioncogenes, and the molecular bases of multistep carcinogenesis. *Cancer Res* **49**: 3713–3721

Xu HJ, Quinlan DC, Davidson AG, Hu SX, Summers CL, Li J and Benedict WF (1994) Altered retinoblastoma protein expression and prognosis in early-stage non-small-cell lung carcinoma. *J Natl Cancer Inst* **86**: 695–699

Yancopoulos GD, Nisen PD, Tesfaye A, Kohl NE, Goldfarb MP and Alt FW (1985) N-*myc* can cooperate with *ras* to transform normal cells in culture. *Proc Natl Acad Sci USA* **82**: 5455–5459

© Cancer Research Campaign 1997